# A comprehensive prognostic and immune infiltration analysis of EXOC3L1 in pan-cancer

**DOI:** 10.3389/fgene.2022.1044100

**Published:** 2022-11-21

**Authors:** Zhan-Fei Zhang

**Affiliations:** ^1^ State Key Laboratory of Oncology in South China, Collaborative Innovation Center for Cancer Medicine, Department of Experimental Research, Sun Yat-Sen University Cancer Center, Guangzhou, China; ^2^ Department of Cardiothoracic Surgery, Zhongshan People’s Hospital, Zhongshan, China

**Keywords:** EXOC3L1, prognosis, immune infiltration, pan-cancer, nomogram

## Abstract

Exocyst complex component 3 like 1 (EXOC3L1) is widely present in various human tissues, which mainly regulates insulin secretion. However, its roles in tumors remain unclear. In the present study, we aimed to investigate the roles of EXOC3L1 in pan-cancer, and the data was downloaded from of the University of California Santa Cruz (UCSC) Xena and the Cancer Genome Atlas (TCGA). The expression status of EXOC3L1 was studied in the TCGA_GTEx samples, TCGA samples and paired samples in TCGA, respectively. Subsequently, Kaplan-Meier analysis was applied to 33 kinds of tumors in TCGA, among the cancers that EXOC3L1 can affect prognosis, clinical correlation analysis and univariate Cox regression analysis were performed. Furthermore, representative cancers kidney renal clear cell carcinoma (KIRC) and lung squamous cell carcinoma (LUSC) with a sample size larger than 500 were selected to construct nomogram models to confirm the prognostic value of EXOC3L1 in cancers. Additionally, the associations of EXOC3L1 with immune cell infiltrations were performed as well. Mechanistically, functional enrichment analysis was performed to explore potential signaling pathways that EXOC3L1 may involve in. Our study found that EXOC3L1 was differentially expressed in a variety of tumors and was associated with the clinical outcomes and immune microenvironment of several tumors, it may affect the occurrence and development of tumors through NOTCH signaling pathway, PI3K-AKT signaling pathway and immune-related pathways. In conclusion, we propose that EXOC3L1 may serve as a potential prognostic biomarker and a promising target for cancer immunotherapy in a variety of cancers.

## Introduction

Worldwide, cancer has seriously endangered public health, and the incidence and mortality of cancer are increasing every year (Bray et al., 2018). Among the most common cancers, lung cancer, breast cancer, esophageal carcinoma, pancreatic cancer and liver cancer have high mortality rate all over the world (Feng et al., 2019). Despite substantial efforts to improve the cancer diagnosis and treatment, the 5-year overall survival (OS) rate for most of the cancers remain dismal (Cao et al., 2021). At the same time, cancers impose a heavy economic burden on countries around the world (Sun et al., 2020). Therefore, there is an urgent need to find new ways to diagnose and treat cancers. At present, the application of cancer biomarkers has made significant progress in some cancers (Zhong et al., 2018; Wu et al., 2020; Bradley et al., 2021; Ren et al., 2021; Tarantino et al., 2022), which has significantly improved the prognosis of indicated tumors. However, we put too much emphasis on individualized precision treatment currently, resulting in the lack of effective biomarkers for many other tumors. Thus, exploring effective biomarkers in pan-cancer can enable many tumors that currently do not have effective targeted drugs to be effectively treated.

Exocyst complex component 3 like 1 (EXOC3L1), also known as EXOC3L, is localized on chromosome 16 (16q22.1). Up to the present day, limited studies have shown that EXOC3L1 were involved in the regulation of insulin secretion (Saito et al., 2008), protein secretion after traumatic brain injury (Tang et al., 2020), spontaneous induction of apoptosis (Matsumoto et al., 2021), and high-density lipoprotein concentration (Lanktree et al., 2015), but the exact function of EXOC3L1 remains unclear. There are no reports on the study of EXOC3L1 in tumors so far, and the roles of EXOC3L1 in pan-cancer are far from clear, making it necessary to explore the role of EXOC3L1 in pan-cancer. Our research aims to investigate the roles of EXOC3L1 in pan-cancer, its correlation with the immune microenvironment, and to preliminarily explore the mechanism of EXOC3L1 in tumors.

In this project, differential expression analysis showed that EXOC3L1 was differentially expressed in most of tumors, and survival analysis showed that EXOC3L1 had an impact on the prognosis of adrenocortical carcinoma (ACC), kidney renal clear cell carcinoma (KIRC), kidney renal papillary cell carcinoma (KIRP), lung squamous cell carcinoma (LUSC), thyroid carcinoma (THCA), pancreatic adenocarcinoma (PAAD). Immune infiltration analysis indicated that the expression of EXOC3L1 was related to various immune cells, and the changes of immune microenvironment could affect the prognosis of tumors. In addition, enrichment analysis suggested that EXOC3L1 may be involved in the occurrence and development of tumors through NOTCH signaling pathway, PI3K-AKT signaling pathway and immune-related pathways. Collectively, EXOC3L1 plays multifaceted roles in pan-cancer, which can affect the prognosis and immune microenvironment of specific cancers, and provides a novel biomarker and a potential therapeutic target.

## Materials and methods

### Data collection and processing

The expression profile data and clinical data of 33 tumors were downloaded from the Cancer Genome Atlas (TCGA, https://portal.gdc.cancer.gov/), while the TCGA_GTEx dataset included TCGA samples and normal samples was collected from the University of California Santa Cruz (UCSC) Xena (https://xenabrowser.net/datapages/). The immunohistochemical images of human normal tissues and tumor tissues were obtained from the Human Protein Atlas (HPA, https://www.proteinatlas.org/). In addition, the Gene Expression Profiling Interactive Analysis database (GEPIA2, http://gepia2.cancer-pku.cn/#index) was used to extract the 100 most relevant genes with EXOC3L1 from the TCGA datasets. This study was in compliance with the published guidelines of TCGA and UCSC, thus, ethical approval and informed consent of the patients were waived.

### Expression analysis of EXOC3L1

The mRNA expression of EXOC3L1 in normal tissues and tumor tissues were compared in TCGA_GTEx samples, TCGA samples and TCGA paired samples, respectively. In addition, the protein level of EXOC3L1 in normal and tumor tissues was investigated in the HPA database.

### Prognosis analysis

Kaplan-Meier analysis with log-rank test was employed to assess the association between EXOC3L1 expression and clinical outcomes including overall survival (OS), progression-free interval (PFI), and disease-specific survival (DSS) in pan-cancer in TCGA, and the survival curves with *p* < 0.05 were shown. Besides, the receiver operating characteristic curve (ROC) were drawn in tumors where EXOC3L1 can affect prognosis.

### Correlation analysis between EXOC3L1 expression and clinical features

The correlation of EXOC3L1 expression with multiple important clinical parameters including gender, T stage, N stage and pathologic stage were investigated in the cancers in which EXOC3L1 can affect prognosis.

### Establishment and evaluation of the nomogram models

In the present study, univariate Cox regression analysis for OS was performed in tumors where EXOC3L1 can affect prognosis, including OS, PFI and DSS, tumors with *p* < 0.05 and sample size greater than 500 were selected to construct a nomogram model respectively, which was an effective and convenient approach for predicting the OS in individual patients. The calibration curves were performed to assess the prediction accuracy of the nomograms at 1-year, 3-year, and 5-year.

### Immune infiltration analysis

Across all the tumors in TCGA, the Tumor Immune Estimation Resource 2.0 (TIMER2.0, http://timer.cistrome.org/) was used to analysis the correlations between EXOC3L1 expression and multiple kinds of immune cells, including B cells, macrophages, T cells CD4^+^, T cells CD8^+^, and a variety of algorithms such as TIMER, EPIC, TIDE, CIBERSORT, CIBERSORT-ABS, QUANTISEQ, XCELL, MCPCOUNTER, etc. Were applied for estimations. More importantly, we further analyzed the effect of immune cells infiltration on OS after stratification of EXOC3L1 in various tumors.

### Functional enrichment analysis and protein-protein interaction network analysis

The 100 EXOC3L1-related genes with most similar expression pattern to EXOC3L1 were obtained from GEPIA2 database. Gene ontology (GO) analysis, which was mainly include biological pathways (BP), and cellular components (CC) and molecular functions (MF), Kyoto Encyclopedia of Genes and Genomes (KEGG) analysis were performed based on the EXOC3L1-related genes to further explore the potential functions of EXOC3L1. In addition, the 100 EXOC3L1-related genes were used to create a PPI network in the Search Tool for the Retrieval of Interacting Genes (STRING) database (https://cn.string-db.org/), and 0.4 was set as the minimum required interaction threshold.

### Differential expression analysis and gene set enrichment analysis

Differential expression analysis of EXOC3L1 was performed in the cancers in which EXOC3L1 can affect the prognosis using the DESeq R package (Love et al., 2014). Subsequently, based on the results obtained from the differential expression analysis of EXOC3L1 in different tumors, GSEA was performed using the clusterProfiler R package (Yu et al., 2012).

### Statistical analysis

The difference between two groups was compared by using the Wilcoxon rank-sum test, while the correlation between two groups was calculated by Spearman rank test. Univariate and multivariate Cox proportional hazard regression were performed to screen the factors influenced the prognosis. Kaplan-Meier analysis with log-rank test was used to survival analysis. Statistical analysis was executed by R (version 4.0.2), and *p* < 0.05 was regarded as statistically significant (*p < 0.05, **p < 0.01, ***p < 0.001, ****p < 0.0001).

## Results

### The expression of EXOC3L1 in pan-cancer

**FIGURE 1:**
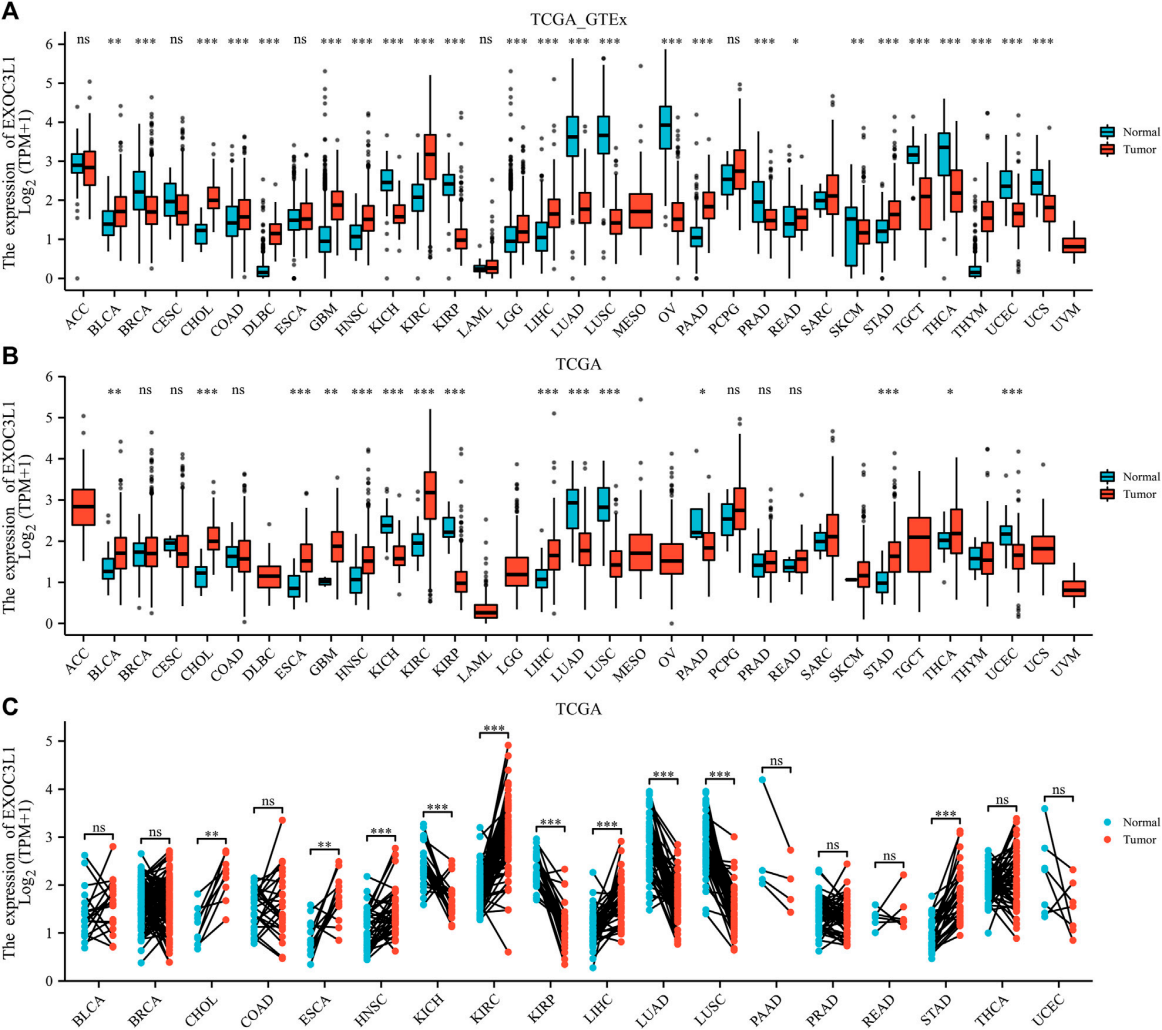
The mRNA expression of EXOC3L1 in pan-cancer. **(A)** The mRNA expression of EXOC3L1 in 33 tumors in TCGA_GTEx samples. **(B)** The mRNA expression of EXOC3L1 in 33 tumors in TCGA database. **(C)** Expression of EXOC3L1 in paired samples of 18 tumors in TCGA database. ACC, adrenocortical carcinoma; BLCA, bladder urothelial carcinoma; BRCA, breast invasive carcinoma; CESC, cervical and endocervical cancers; CHOL, cholangiocarcinoma; COAD, colon adenocarcinoma; DLBC, lymphoid neoplasm diffuse large B-cell lymphoma; ESCA, esophageal carcinoma; GBM, glioblastoma multiforme; HNSC, head and neck squamous cell carcinoma; KICH, kidney chromophobe; KIRC, kidney renal clear cell carcinoma; KIRP, kidney renal papillary cell carcinoma; LAML, acute myeloid leukemia; LGG, brain lower grade glioma; LIHC, liver hepatocellular carcinoma; LUAD, lung adenocarcinoma; LUSC, lung squamous cell carcinoma; MESO, mesothelioma; OV, ovarian serous cystadenocarcinoma; PAAD, pancreatic adenocarcinoma; PCPG, pheochromocytoma and paraganglioma; PRAD, prostate adenocarcinoma; READ, rectum adenocarcinoma; SARC, sarcoma; SKCM, skin cutaneous melanoma; STAD, stomach adenocarcinoma; STES, stomach and esophageal carcinoma; TGCT, testicular germ cell tumors; THCA, thyroid carcinoma; THYM, thymoma; UCEC, uterine corpus endometrial carcinoma; UCS, uterine carcinosarcoma; UVM, uveal melanoma. (ns, *p* > 0.05; *p < 0.05; **p < 0.01; ***p < 0.001).

**FIGURE 2:**
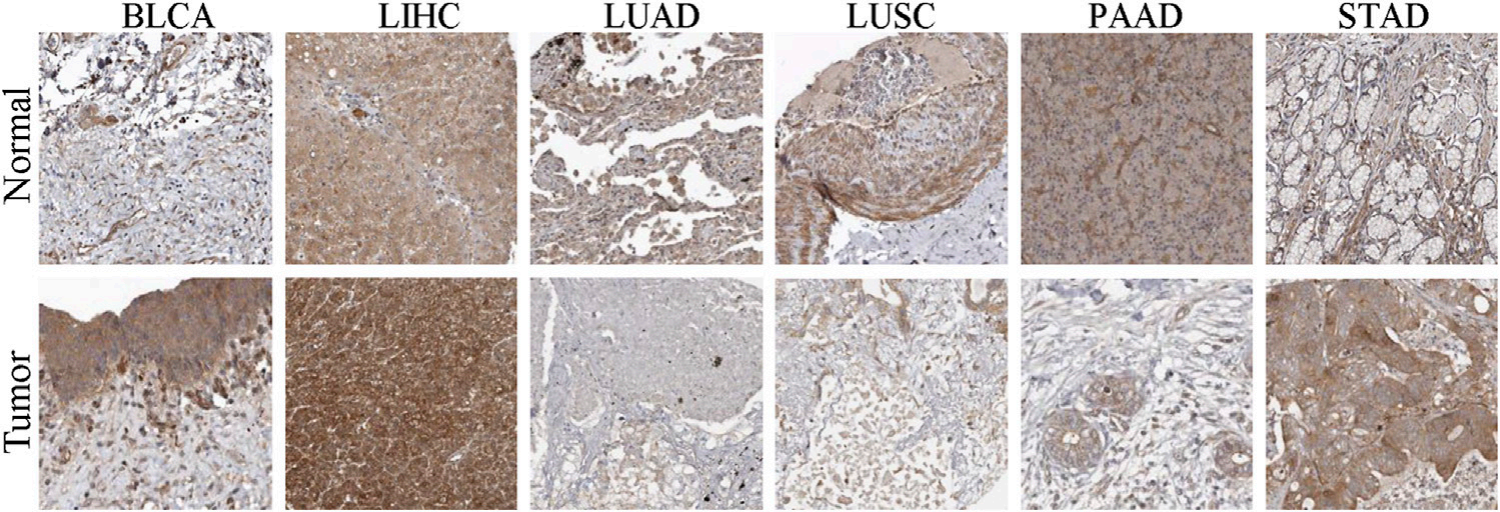
The IHC images of EXOC3L1 in normal and tumor tissues extracted from the HPA.

In order to clarify the expression of EXOC3L1 in pan-cancer, the uniformed TCGA_GTEx data collected from the UCSC were analyzed. The results showed that EXOC3L1 was differentially expressed in most tumors, some with high expression and some with low expression (Figure 1A), which was roughly in agreement with the results obtained from TCGA (Figure 1B), additionally, the expression of EXOC3L1 in 18 kinds of tumors with paired samples in TCGA was also analyzed (Figure 1C). Further, we studied the protein expression of EXOC3L1 in normal tissues and tumor tissues of different human organs on the HPA, and representative immunohistochemical (IHC) images of normal and tumor tissues of bladder, liver, lung, pancreas, and stomach were extracted (Figure 2).

### The association between EXOC3L1 expression and prognosis in pan-cancer

**FIGURE 3:**
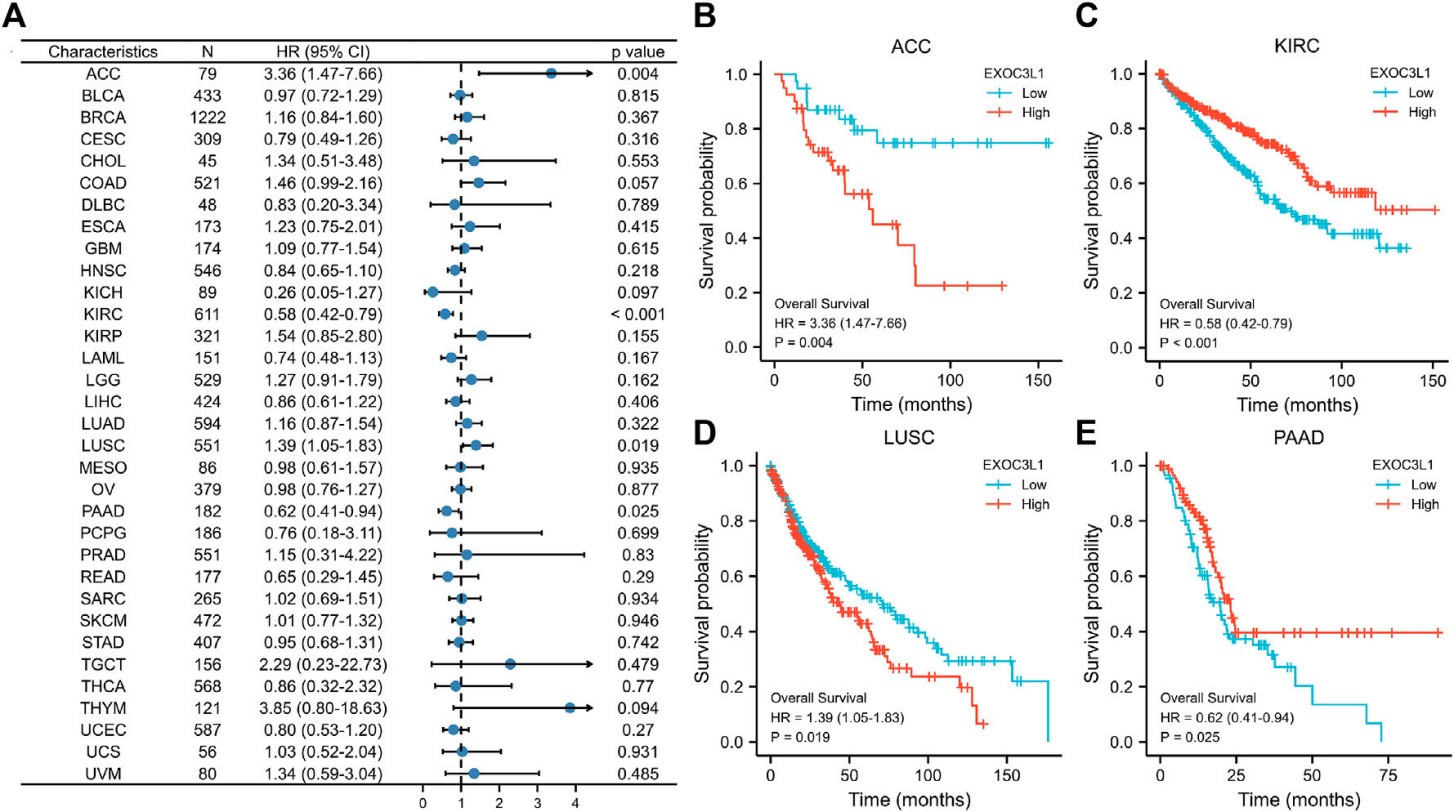
The association between EXOC3L1 expression and OS in pan-cancer. **(A)** The effects of EXOC3L1 expression on OS in pan-cancer were exhibited by a forest plot. **(B–E)** Effects of EXOC3L1 expression on OS in ACC, KIRC, LUSC and PAAD, respectively.

In order to survey the prognostic assessment value of EXOC3L1 in pan-cancer, Kaplan-Meier survival analysis was carried out to evaluate the relationship between EXOC3L1 expression and clinical outcomes. First of all, we investigated the relationship between the EXOC3L1 expression and OS in 33 cancers (Figure 3A), and the results pointed out that the aberrant expression of EXOC3L1 was correlated with OS in ACC (Figure 3B), KIRC (Figure 3C), LUSC (Figure 3D) and PAAD (Figure 3E), high expression of EXOC3LI was correlated with shorter OS in ACC and LUSC, whereas high expression of EXOC3L1 in KIRC and PAAD meant longer OS.

**FIGURE 4:**
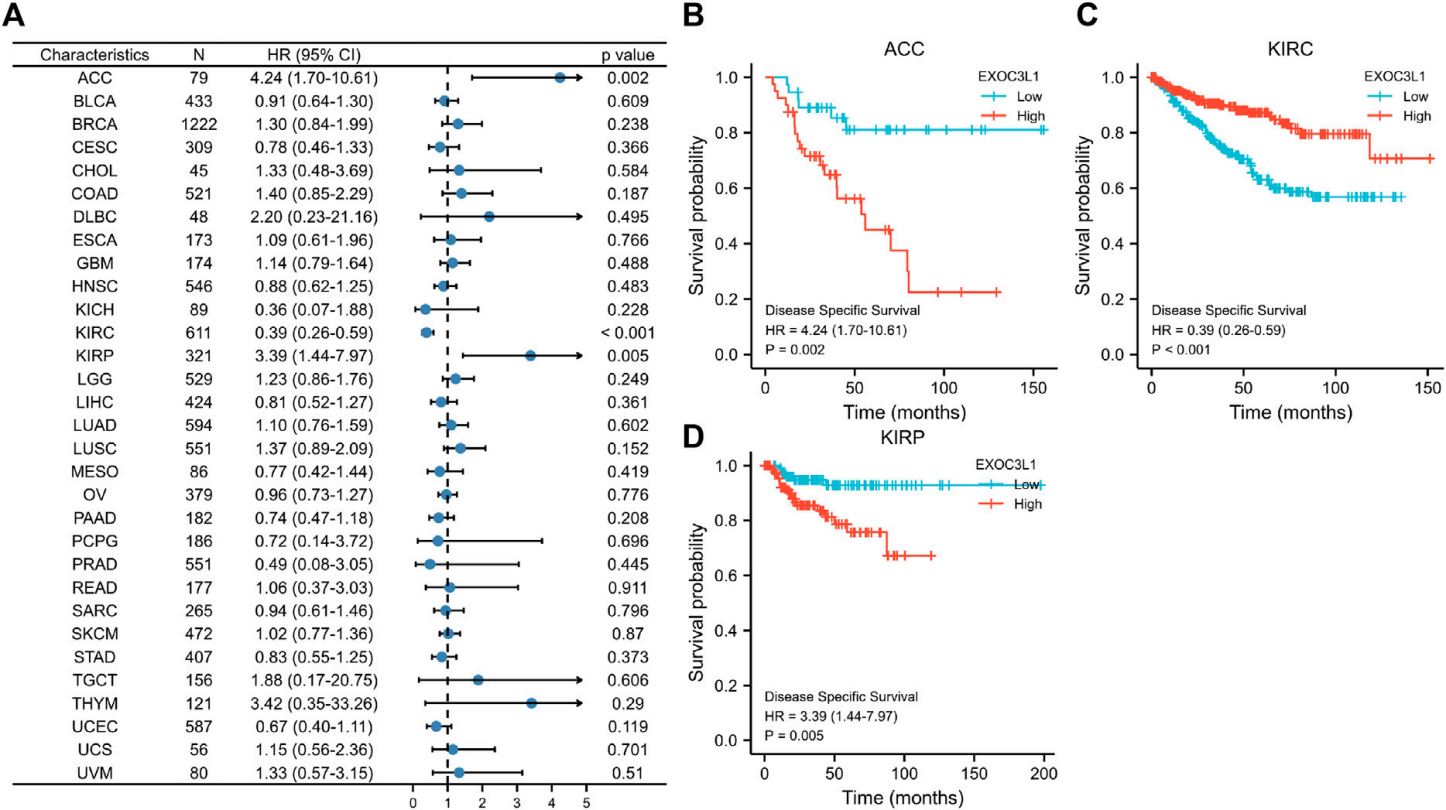
The relationship between EXOC3L1 expression and DSS in pan-cancer. **(A)** The associations of EXOC3L1 expression on DSS in various cancers were displayed by a forest diagram. **(B–D)** Survival curves of EXOC3L1 on DSS in ACC, KIRC and KIRP, respectively.

Next, we investigated the relationship between the expression of EXOC3L1 and DSS (Figure 4A), and the results suggested that the expression of EXOC3L1 was related to the DSS of ACC (Figure 4B), KIRC (Figure 4C), and KIRP (Figure 4D). In ACC and KIRP, EXOC3L1 high expression was associated with worse DSS, while in KIRC predicted better DSS.

**FIGURE 5:**
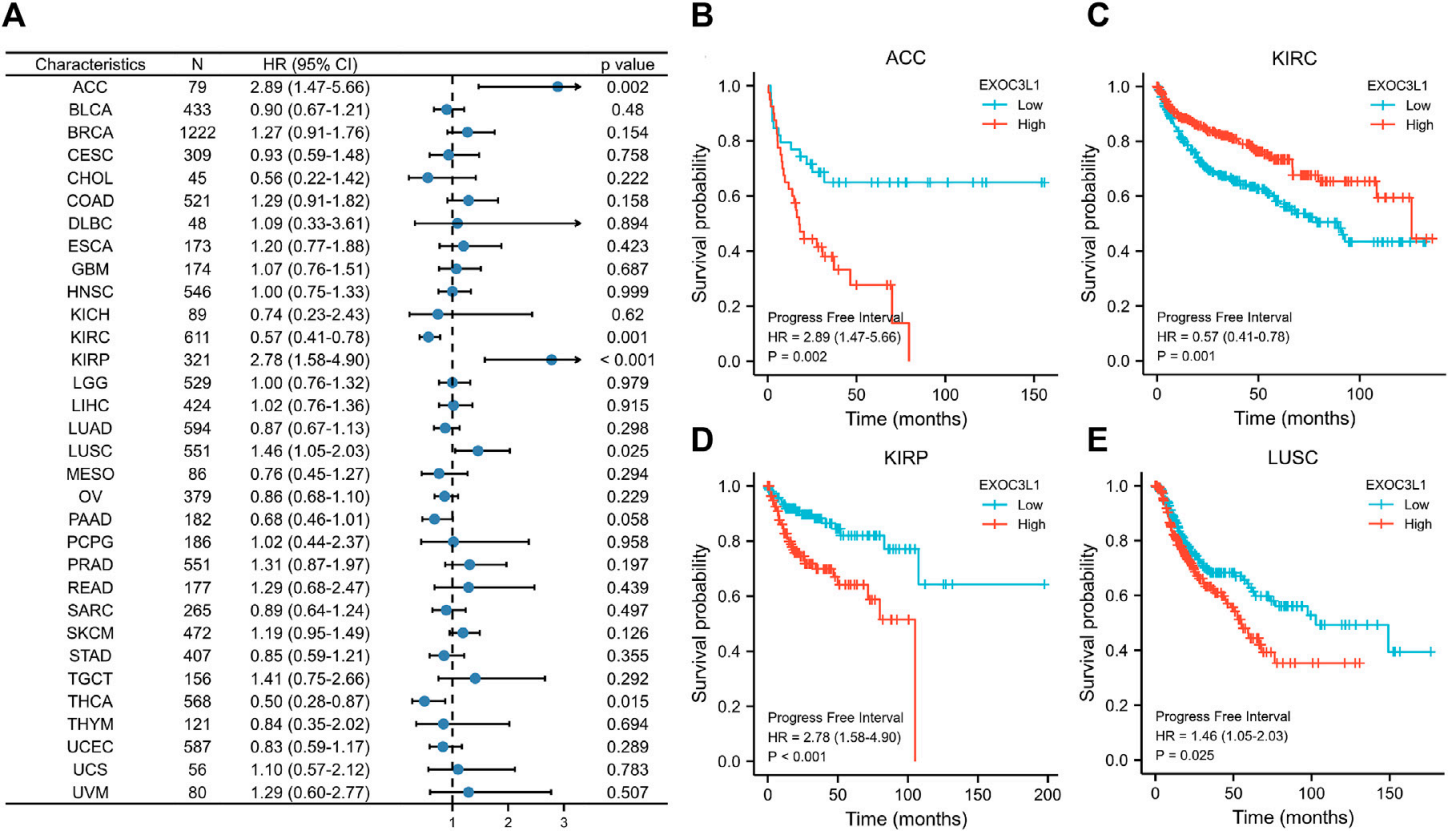
Effect of EXOC3L1 expression on PFI in pan-cancer. **(A)** A forest map of the relationship of EXOC3L1 expression on PFI in TCGA. **(B–E)** Effects of EXOC3L1 expression on PFI in ACC, KIRC, KIRP and LUSC, respectively.

Finally, we explored the relationship between the expression of EXOC3L1 and PFI (Figure 5A), the results showed that the expression of EXOC3L1 was related to the PFI of ACC (Figure 5B), KIRC (Figure 5C), KIRP (Figure 5D), LUSC (Figure 5E), and high expression of EXOC3L1 represents worse PFI in ACC, KIRP and LUSC, but better in KIRC. In addition, the ROC curves of six tumors whose prognosis was associated with EXOC3L1 expression were also presented (Supplementary Figures S1A–F), representing the diagnostic ability of EXOC3L1 in these tumors.

### The correlations between EXOC3L1 expression and clinical parameters

**FIGURE 6:**
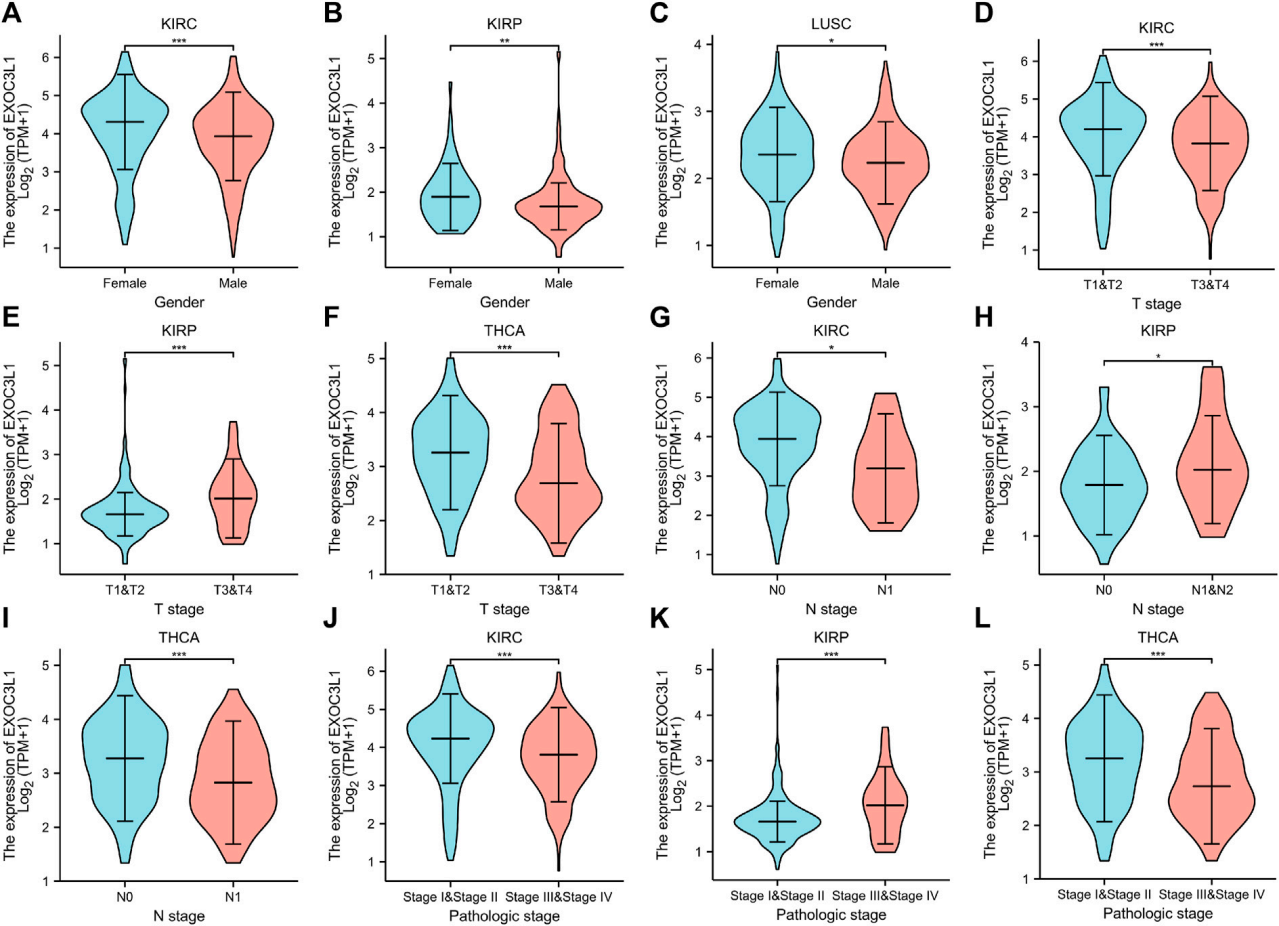
The correlation between EXOC3L1 expression and clinicopathological parameters. **(A–C)** EXOC3L1 expression was correlated with gender in KIRC, KIRP and LUSC. **(D–F)** Expression of EXOC3L1 was correlated with T stage in KIRC, KIRP and THCA. **(G–I)** EXOC3L1 expression was associated with N stage in KIRC, KIRP and THCA. **(J–L)** EXOC3L1 expression was associated with pathologic stage in KIRC, KIRP and THCA. (*p < 0.05; **p < 0.01; ***p < 0.001).

Among the 33 tumors in TCGA database, the prognosis of six tumors including ACC, KIRC, KIRP, LUSC, PAAD, THCA were associated with the expression of EXOC3L1. Here, we studied the association between EXOC3L1 expression and clinicopathological features in these six tumors, and the results suggested that EXOC3L1 expression was correlated with gender in KIRC, KIRP and LUSC (Figures 6A–C). Meanwhile, the expression of EXOC3L1 in KIRC, KIRP and THCA was correlated with tumor size (Figures 6D–F), lymph node metastasis (Figures 6G–I) and pathological stage (Figures 6J–L).

### Construction and evaluation of nomogram models in kidney renal clear cell carcinoma and lung squamous cell carcinoma

**FIGURE 7:**
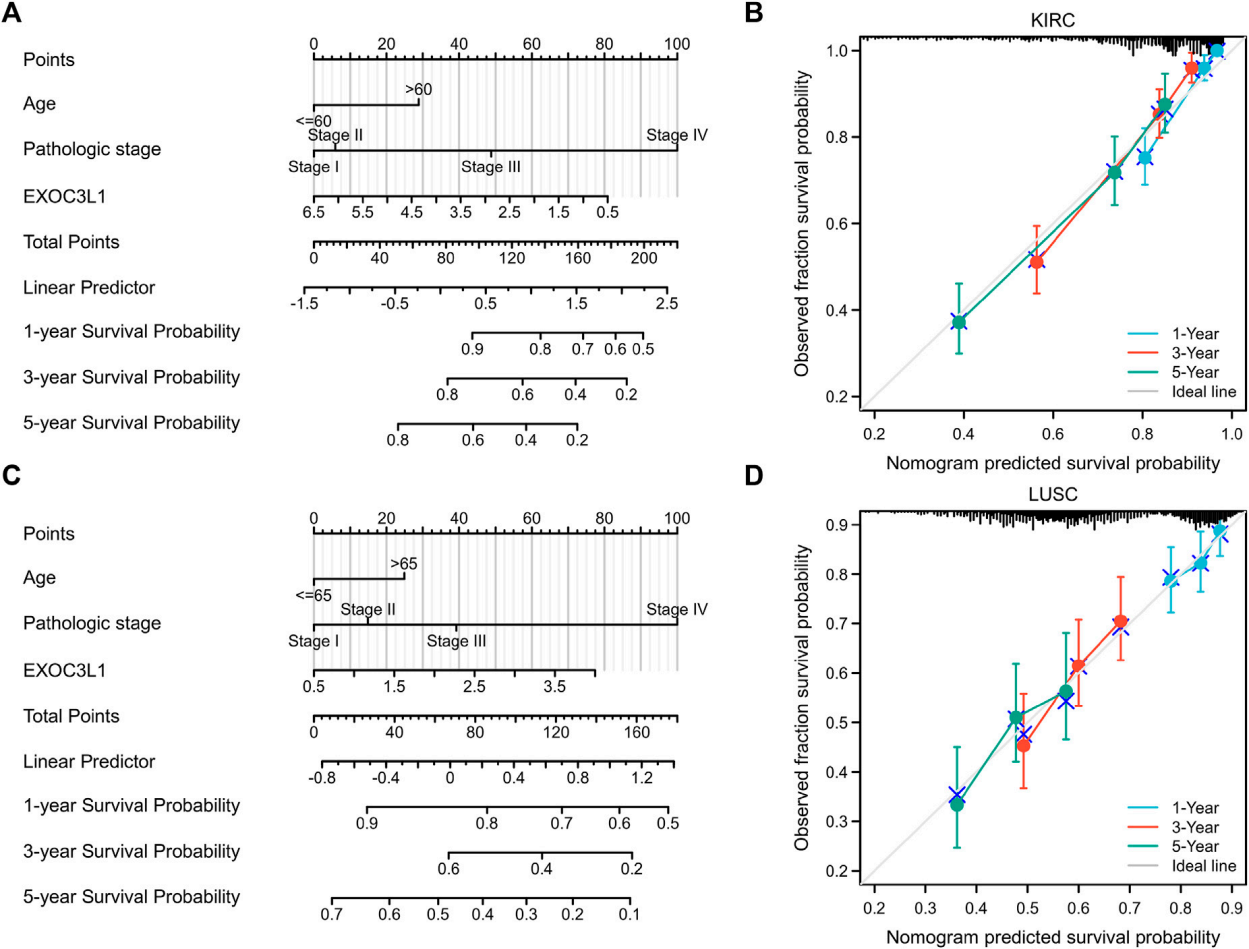
Nomogram models were established and evaluated in KIRC and LUSC. **(A)** Establishment of a nomogram model incorporating EXOC3L1 expression in KIRC. **(B)** Calibration curves were used to evaluate the nomogram model in KIRC at 1-year, 3-year, and 5-year. **(C)** Building a nomogram model containing EXOC3L1 expression in LUSC. **(D)** The 1-year, 3-year and 5-year calibration curves were used to evaluate the prediction accuracy of the nomogram model in LUSC.

To further investigate the effect of EXOC3L1 expression on the prognosis of specific cancers, we performed univariate Cox regression analysis for OS in six tumors in which EXOC3L1 can affect the prognosis (Supplementary Tables S1–S6). Based on the results of univariate Cox regression, LUSC and KIRC with a sample size greater than 500 were selected to construct a nomogram model to verify the prognosis value respectively, and the calibration curves were used to evaluate the prediction accuracy of the nomogram model at 1-year, 3-year, and 5-year. The results showed that in the nomogram models, EXOC3L1 contributed significantly to the prognosis and exhibited good predictive power for the OS of KIRC (Figure 7A) and LUSC (Figure 7C), and the 1-year, 3-year, 5-year survival prediction curves of the calibration suggested that the nomogram models had a high accuracy in predicting OS (Figures 7B,D).

### The correlation of EXOC3L1 expression and tumor immune microenvironment

**FIGURE 8:**
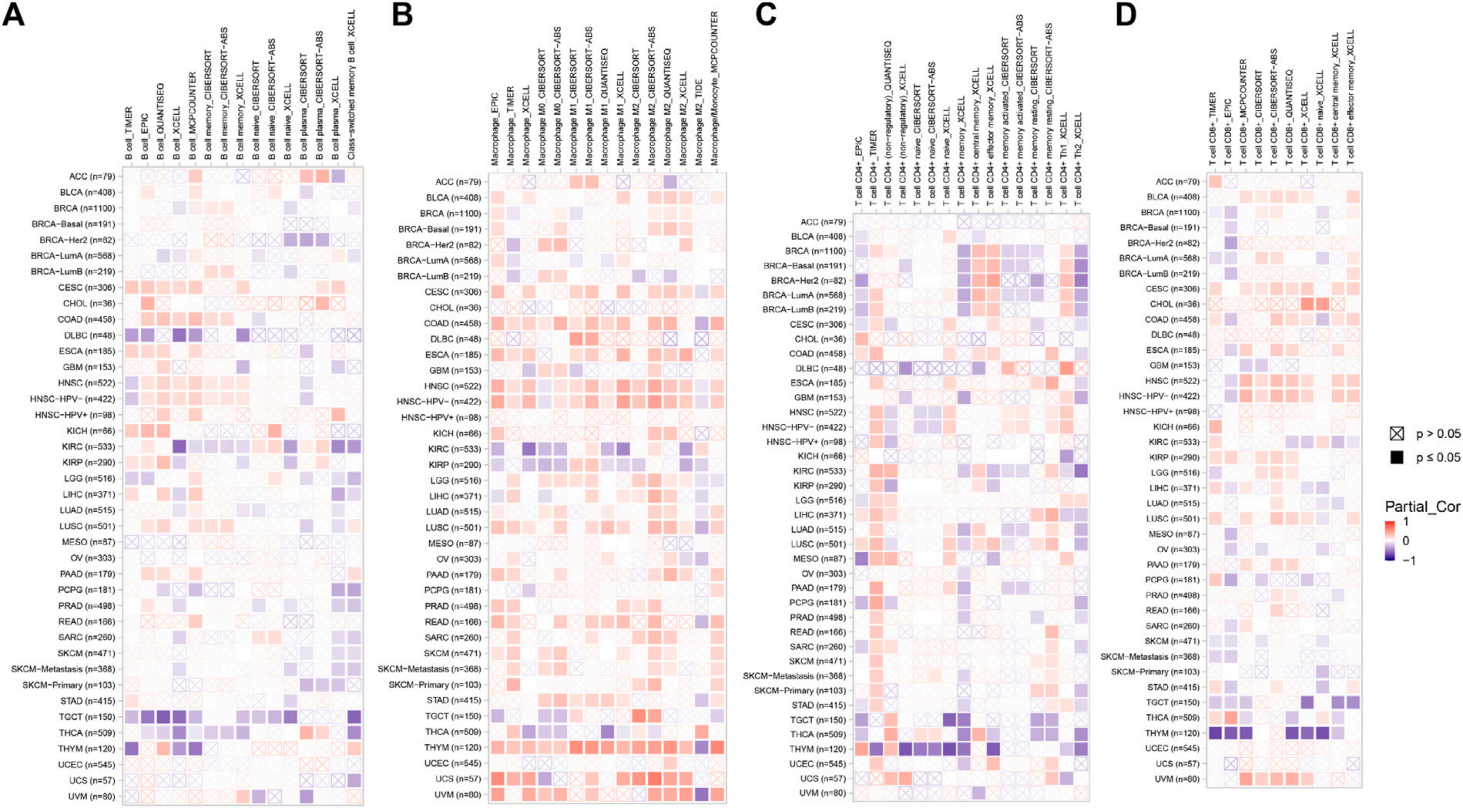
The correlation of EXOC3L1 expression and immune cell infiltration. **(A–D)** Heatmaps of correlations between EXOC3L1 expression and B cells, macrophages, T cell CD4^+^, and T cell CD8^+^ in TIMER2 database, respectively.

Immune microenvironment plays a crucial role in the occurrence and development of tumors. In order to study the relationship between EXOC3L1 and immune microenvironment in pan-cancer, the correlation between EXOC3L1 expression and immune cells in pan-cancer was carried out by using the GEPIA2 database. The heatmaps of the correlation between EXOC3L1 expression and B cells (Figure 8A), macrophages (Figure 8B), T cells CD4^+^ (Figure 8C), and T cells CD8^+^ (Figure 8D) were shown.

### Effect of EXOC3L1 expression combined with immune infiltration on overall survival

**FIGURE 9:**
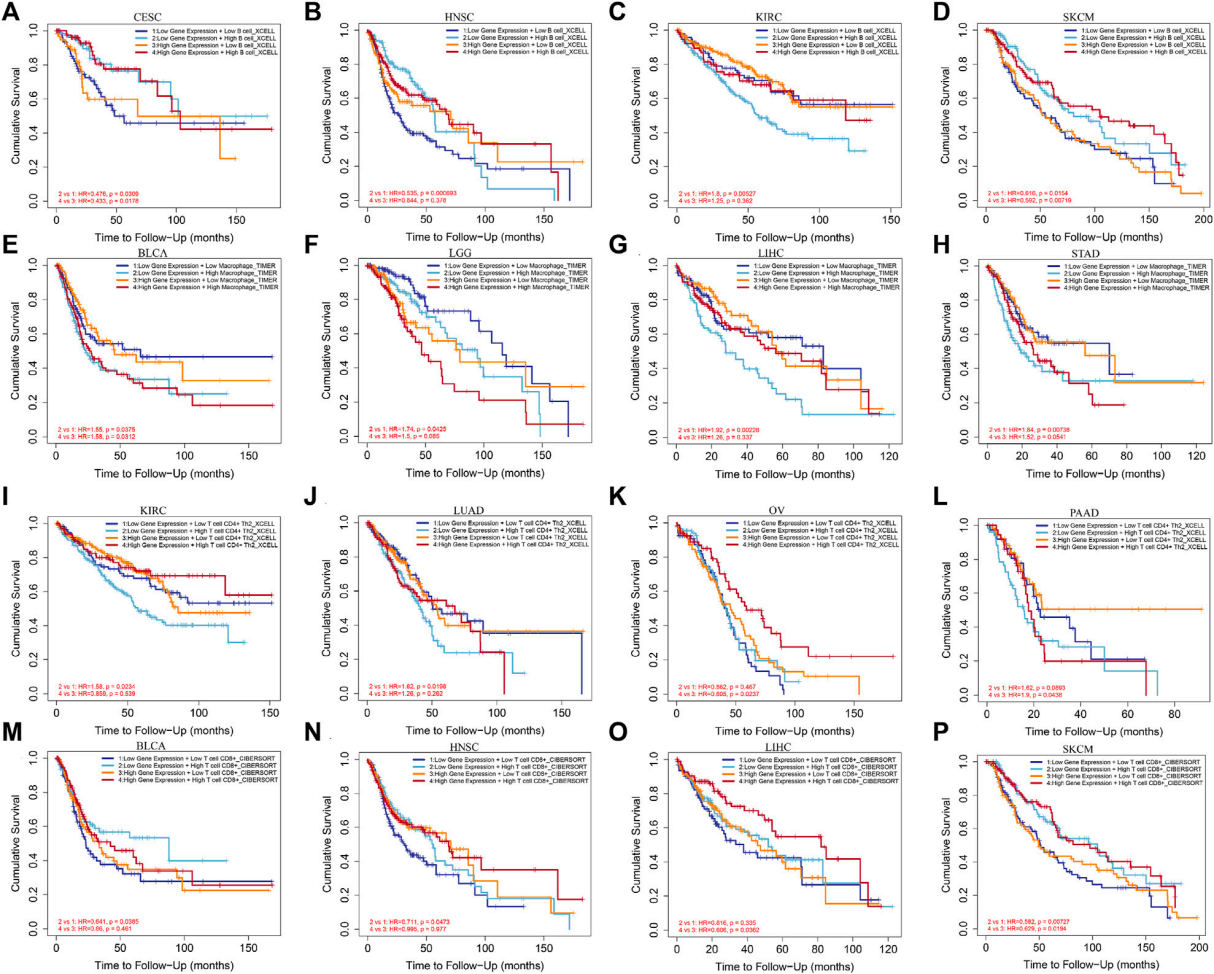
The effect of immune cells infiltration on OS was related to the expression of EXOC3L1. **(A–D)** Effect of B cells infiltration on OS of CESC, HNSC, KIRC and SKCM at different EXOC3L1 expression levels. **(E–H)** Effect of macrophages infiltration on OS of BLCA, LGG, LIHC and STAD at different EXOC3L1 expression levels. **(I–L)** Effect of T cell CD4^+^ expression on OS of KIRC, LUAD, OV, and PAAD at different EXOC3L1 expression levels. **(M–P)** Effect of T cell CD8^+^ expression on OS of BLCA, HNSC, LIHC and SKCM at different EXOC3L1 expression levels.

In order to detect the impact of immune cells infiltration on tumor prognosis, we combined the expression of EXOC3L1 and immune cell infiltration to analyze the effect on tumor OS in GEPIA2. The results suggested that B cells infiltration has an impact on the prognosis of CESC, HNSC, KIRC, and SKCM (Figures 9A–D). Macrophages infiltration was associated with OS in BLCA, LGG, LIHC and STAD (Figures 9E–H). T cells CD4^+^ infiltration was associated with OS in KIRC, LUAD, OV, and PAAD (Figures 9I–L). T cells CD8^+^ infiltration was associated with the prognosis of BLCA, HNSC, LIHC, and SKCM (Figures 9M–P). More importantly, in different expression levels of EXOC3L1, immune cells have different effects on tumor prognosis, suggesting that the function of immune cells may depend on the expression level of EXOC3L1.

### Functional enrichment analysis and protein-protein interaction analysis of EXOC3L1-related genes

**FIGURE 10:**
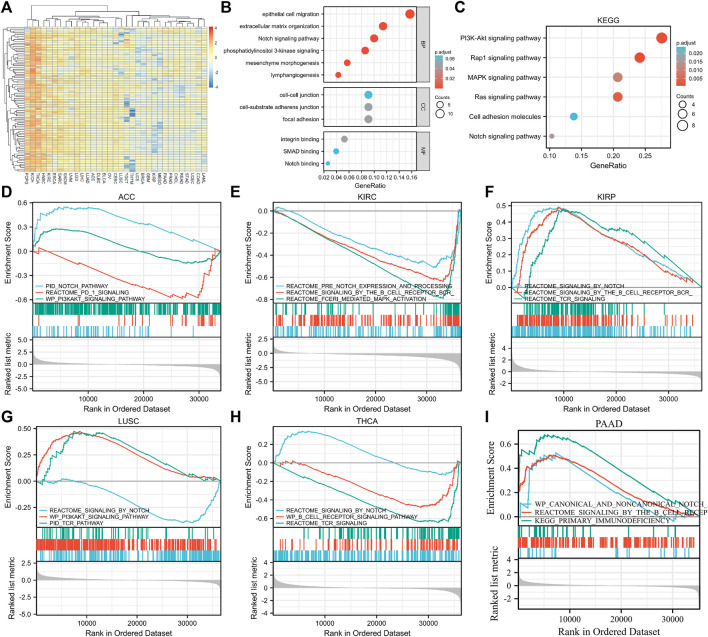
Functional enrichment analysis of EXOC3L1-related genes. **(A)** Heatmap of EXOC3L1-related genes expression in 33 tumors in TCGA database. **(B)** GO enrichment analysis based on 100 EXOC3L1-related genes, including BP, CC, and MF. **(C)** KEGG pathways analysis based on 100 EXOC3L1-related genes. **(D–I)** GSEA based on the differentially expression analysis in ACC, KIRC, KIRP, LUSC, THCA, and PAAD, respectively.

To further elucidate the biological function of EXOC3L1 in tumors, 100 genes most related to EXOC3L1 were obtained from GEPIA2 database (Supplementary Table S7), and an expression heatmap of EXOC3L1-related genes in 33 tumors was shown in Figure 10A. GO analysis (Figure 10B) suggested that EXOC3L1-related genes may participate in the “epithelial cell migration”, “extracellular matrix organization”, “Notch Signaling pathway”, “PI3K signaling”, “mesenchyme morphogenesis”, “lymphangiogenesis” and other biological processes. Involved in “cell-cell junction”, “cell-substrate adherens junction”, “focal adhesion” and other cell components. It participates in “integrin binding”, “SMAD binding”, “Notch binding” and other molecular functions. KEGG pathway analysis (Figure 10C) indicated that EXOC3L1-related genes may be related to “PI3K-AKT signaling pathway”, “Rap1 signaling pathway”, “MAPK signaling pathway”, “Ras signaling pathway”, “Cell adhesion molecules”, and “Notch signaling pathway”. Additionally, 100 EXOC3L1-related genes were used to generate a PPI network on the STRING website (Supplementary Figure S2).

### Gene set enrichment analysis

To further determine the function of EXOC3L1, the GSEA based on the differential expression analysis of EXOC3L1 was applied to elucidate the biological function of EXOC3L1 in the six tumors whose EXOC3L1 expression was associated with prognosis, including ACC (Figure 10D), KIRC (Figure 10E), KIRP (Figure 10F), LUSC (Figure 10G), THCA (Figure 10H), PAAD (Figure 10I). The results suggest that EXOC3L1 was mainly related to immune-related pathways, Notch pathway and PI3K-AKT signaling pathway.

## Discussion

Tumors are serious threat to human lives. Although effective biomarkers have been found in some tumors, there are still many tumors that lack of effective diagnostic and therapeutic targets (Grossberg et al., 2020; DiSiena et al., 2021), resulting in very unsatisfactory prognosis. Therefore, mining biomarkers that are effective for a variety of tumors can allow cancers that have no effective targets to be effectively treated.

This study unveiled that EXOC3L1 could affect the prognosis of some tumors and was correlated with a variety of immune cells. Notably, the changes of immune cells infiltration can affect the prognosis of various tumors, more importantly, our finding demonstrated that the function of immune cells may depend on the expression of EXOC3L1. In addition, enrichment analysis suggested that EXOC3L1 may affect tumor progression through NOTCH signaling pathway, PI3K-AKT and immune-related pathways.

At present, there is a lack of research on EXOC3L1 in pan-cancer. Therefore, the expression status of EXOC3L1 in different cancers is still unclear. Here, for the first time, we explore the relationship between EXOC3L1 and pan-cancer. We studied the expression differences of EXOC3L1 between normal tissues and tumor tissues of different organs in TCGA_GTEx samples, TCGA samples and TCGA paired samples respectively. In general, three different datasets obtained generally consistent conclusions, but there were also inconsistent or even opposite results, such as the results of PAAD obtained in TCGA_GTEx dataset and TCGA dataset were opposite, which was caused by the difference of sample size in the control group. Thus, it is necessary to increase the sample size of the control group in order to get more accurate conclusion.

Nowadays more and more studies have shown that immune cell infiltration has an impact on the prognosis of tumors (Tosolini et al., 2011; Gajewski et al., 2013; Fu and Jiang, 2018; Grisaru-Tal et al., 2020). Similarly, our study also showed that the expression level of immune cells was correlated with the prognosis of tumors in different cancers. However, different from many studies, we divided the expression of EXOC3L1 into high expression group and low expression group, and then evaluated the effect of the expression of immune cells on OS, which made it easier to find the effect of EXOC3L1 on the immune cells. Based on these results, we speculate that the effect of immune cells on tumor OS depend on the expression level of EXOC3L1. Differences in gene expression leads to the changes in the immune microenvironment, providing great potential for tumor immunotherapy (Leko and Rosenberg, 2020; Murciano-Goroff et al., 2020; Bejarano et al., 2021; Grisaru-Tal et al., 2022; Mantovani et al., 2022), which has made great progress in several researches (Luo et al., 2021; Sangro et al., 2021; Doki et al., 2022; Powles et al., 2022). This study revealed that EXOC3L1 could regulate the immune microenvironment, suggesting that EXOC3L1 may be applied in the development of new-targeted drugs for immunotherapy for some cancers, and benefit a large number of patients suffering cancers.

Likewise, the clinical relevance of EXOC3L1 to tumors and prognosis have not been investigated. Our study found that EXOC3L1 was related to the N stage of some tumors, which prompted us to wonder whether EXOC3L1 plays a role in lymph node metastasis, and the results were in line with the enrichment analysis items of “epithelial cell migration”, “extracellular matrix organization”, “mesenchyme morphogenesis”, “lymphangiogenesis”, “cell-cell junction”, “cell-substrate adherens junction”, “focal adhesion”, “integrin Binding”, and “SMAD binding”, suggesting that EXOC3L1 may be an important target for controlling lymph node metastasis in specific tumors. In addition, it is worth noting that EXOC3L1 is lowly expressed in LUSC and KIRP, but it plays the role of an oncogene, and is highly expressed in KIRC, but functions as a tumor suppressor gene, this complex relationship requires more experiments to further explain.

The following are the advantages of this study. Firstly, we were firstly studied the role and mechanism of EXOC3L1 in pan-cancer, and elaborated on expression differences, clinical correlation analysis, survival analysis, immune infiltration analysis, enrichment analysis, etc., which deepened our understanding of EXOC3L1. Secondly, on the basis of survival analysis, we selected representative cancers KIRC and LUSC with large sample size to further clarify that EXOC3L1 can have an impact on the prognosis of tumors, which makes the conclusions more reliable. Thirdly, EXOC3L1 combined with immune cells was used to evaluate the prognosis of tumors, suggesting that the function of immune cells may depend on the expression of EXOC3L1, which further clarified the relationship between EXOC3L1, tumor immune cells and prognosis. Lastly, this study revealed that EXOC3L1 may be involved in tumor development through NOTCH signaling, PI3K-AKT signaling and immune-related pathways, providing directions for future mechanistic studies.

Although we did our best to make the results of our research accurate and reliable, some limitations were inevitable. On one hand, the sample size of some cancers was small or the sample size of the control group was insufficient, resulting in ambiguous conclusions. On the other hand, this study mainly focuses on bioinformatics analysis, and the conclusions obtained need to be confirmed by further *in vivo* and *in vitro* experiments.

In summary, we found that EXOC3L1 was related to the prognosis and exerts its function via the regulation of immune microenvironment in a variety of tumors. Mechanistically, it may affect the initiation and development of tumors through NOTCH signaling pathway, PI3K-AKT signaling pathway and immune-related pathways. However, the specific roles and mechanisms still need further experimental verification.

## Data Availability

Publicly available datasets were analyzed in this study, and the data can be found in the TCGA database and UCSC database.
