# Supplementary material for: A comprehensive prognostic and immune infiltration analysis of EXOC3L1 in pan-cancer
Source: Front Genet. 2022 Nov 21;13:1044100. doi: 10.3389/fgene.2022.1044100 (PMC9720260; doi:10.3389/fgene.2022.1044100)
Supplement: Supplementary file 1 [file DataSheet1.docx]

Supplementary Material

# Supplementary Figures

**
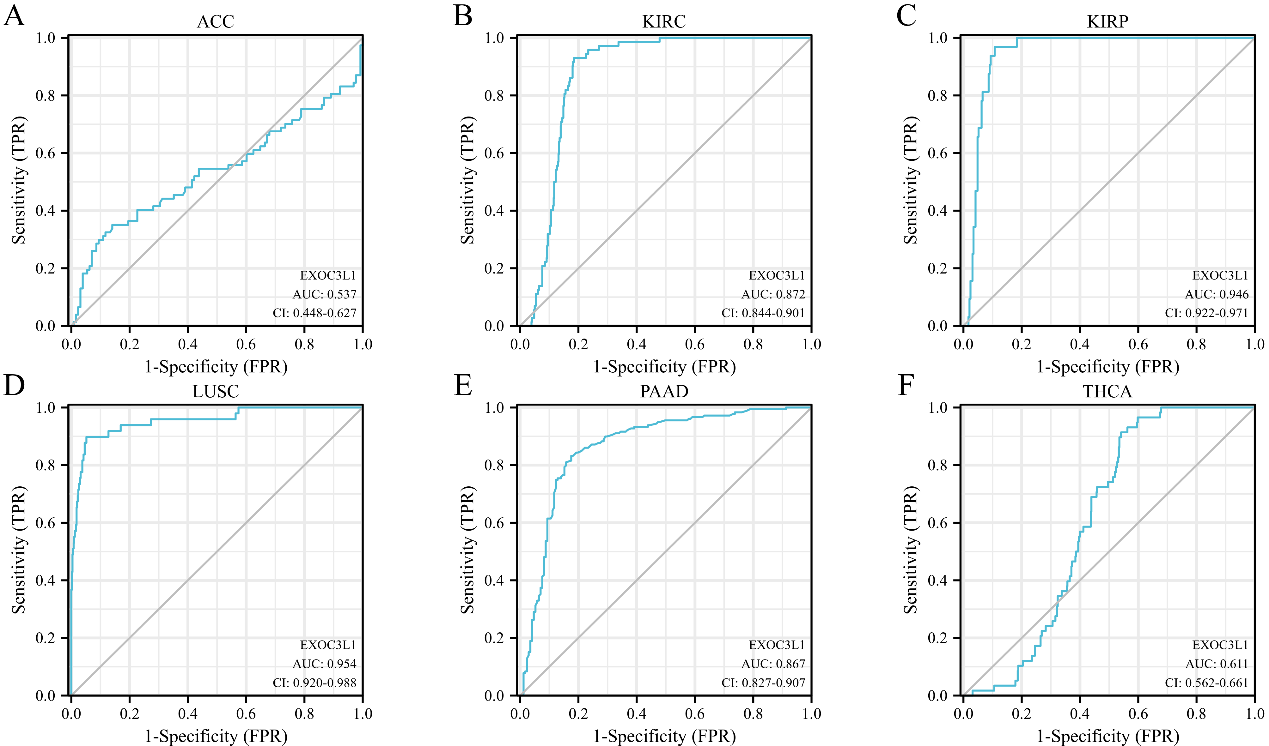
**

**Supplementary Figure S1. ROC curves of KIRC, KIRP, LUSC and PAAD in TCGA database.**

**
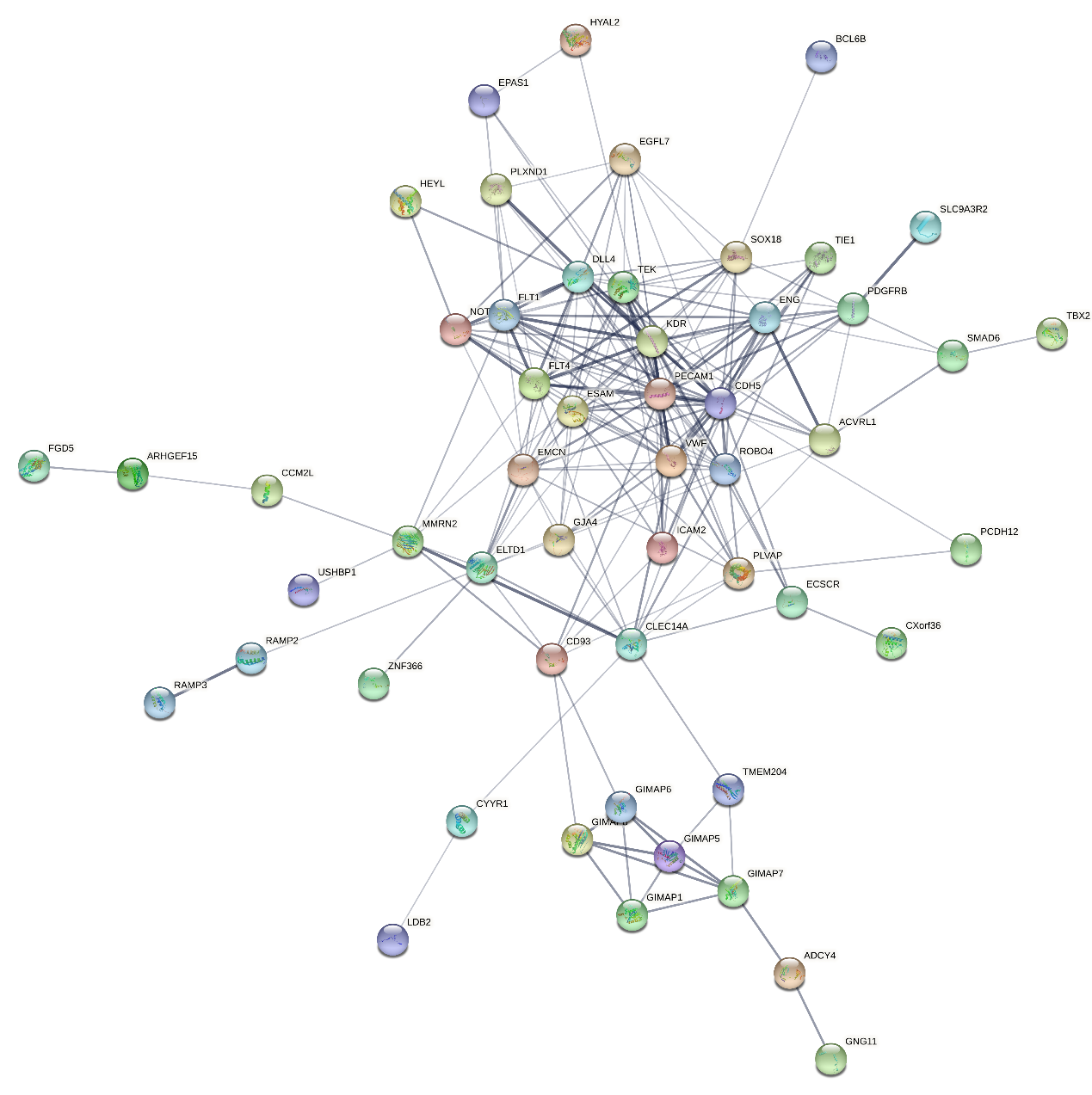
**

**Supplementary Figure S2. Diagram of protein-protein interaction network of 100 EXOC3L1-related genes.**

# Supplementary Tables

**Table S1.** Univariate and multivariate Cox regression analysis for OS in ACC.

| Characteristics | N | Univariate analysis | |  | Multivariate analysis | |
| --- | --- | --- | --- | --- | --- | --- |
|  |  | Hazard ratio (95% CI) | p |  | Hazard ratio (95% CI) | p |
| **Age** | 79 |  |  |  |  |  |
| ≤50 | 41 | Reference |  |  |  |  |
| >50 | 38 | 1.799 (0.846-3.824) | 0.127 |  |  |  |
| **Gender** | 79 |  |  |  |  |  |
| Female | 48 | Reference |  |  |  |  |
| Male | 31 | 1.001 (0.469-2.137) | 0.999 |  |  |  |
| **Tumor status** | 77 |  |  |  |  |  |
| Tumor free | 39 | Reference |  |  |  |  |
| With tumor | 38 | 1454360405.063 (0.000-Inf) | 0.997 |  |  |  |
| **New event** | 76 |  |  |  |  |  |
| No | 39 | Reference |  |  |  |  |
| Yes | 37 | 10.238 (3.050-34.359) | **<0.001** |  | 5.706 (1.611-20.214) | **0.007** |
| **Pathologic stage** | 77 |  |  |  |  |  |
| I-II | 46 | Reference |  |  |  |  |
| III-IV | 31 | 6.476 (2.706-15.498) | **<0.001** |  | 2.983 (1.221-7.287) | **0.016** |
| **Laterality** | 79 |  |  |  |  |  |
| Left | 45 | Reference |  |  |  |  |
| Right | 34 | 0.841 (0.394-1.796) | 0.655 |  |  |  |
| **EXOC3L1** | 79 |  |  |  |  |  |
| Low | 39 | Reference |  |  |  |  |
| High | 40 | 3.360 (1.473-7.662) | **0.004** |  | 1.912 (0.744-4.919) | 0.179 |

**Table S2.** Univariate and multivariate Cox regression analysis for OS in KIRC.

| Characteristics | N | Univariate analysis | |  | Multivariate analysis | |
| --- | --- | --- | --- | --- | --- | --- |
|  |  | Hazard ratio (95% CI) | p |  | Hazard ratio (95% CI) | p |
| **Age** | 539 |  |  |  |  |  |
| ≤60 | 269 | Reference |  |  |  |  |
| >60 | 270 | 1.765 (1.298-2.398) | **<0.001** |  | 1.538 (1.128-2.096) | **0.006** |
| **Gender** | 539 |  |  |  |  |  |
| Female | 186 | Reference |  |  |  |  |
| Male | 353 | 0.930 (0.682-1.268) | 0.648 |  |  |  |
| **Race** | 532 |  |  |  |  |  |
| Asian | 8 | Reference |  |  |  |  |
| White | 467 | 1.833 (0.256-13.125) | 0.546 |  |  |  |
| Others | 57 | 1.572 (0.203-12.187) | 0.665 |  |  |  |
| **Pathologic stage** | 536 |  |  |  |  |  |
| I-II | 331 | Reference |  |  |  |  |
| III-IV | 205 | 3.946 (2.872-5.423) | **<0.001** |  | 2.635 (1.857-3.738) | **<0.001** |
| **Histologic grade** | 531 |  |  |  |  |  |
| G1 | 14 | Reference |  |  |  |  |
| G2 | 235 | 7510356.751 (0.000-Inf) | 0.994 |  | 5872084.591 (0.000-Inf) | 0.994 |
| G3 | 207 | 14161426.542 (0.000-Inf) | 0.993 |  | 8318697.441 (0.000-Inf) | 0.994 |
| G4 | 75 | 38204822.146 (0.000-Inf) | 0.993 |  | 15585177.927 (0.000-Inf) | 0.993 |
| **EXOC3L1** | 539 |  |  |  |  |  |
| Low | 269 | Reference |  |  |  |  |
| High | 270 | 0.578 (0.425-0.785) | **<0.001** |  | 0.886 (0.636-1.232) | 0.471 |

**Table S3.** Univariate and multivariate Cox regression analysis for OS in LUSC.

| Characteristics | N | Univariate analysis | |  | Multivariate analysis | |
| --- | --- | --- | --- | --- | --- | --- |
|  |  | Hazard ratio (95% CI) | p |  | Hazard ratio (95% CI) | p |
| **Age** | 490 |  |  |  |  |  |
| ≤65 | 190 | Reference |  |  |  |  |
| >65 | 300 | 1.279 (0.960-1.704) | 0.093 |  | 1.361 (1.018-1.820) | **0.038** |
| **Gender** | 496 |  |  |  |  |  |
| Female | 130 | Reference |  |  |  |  |
| Male | 366 | 1.211 (0.879-1.669) | 0.241 |  |  |  |
| **Smoker** | 484 |  |  |  |  |  |
| No | 18 | Reference |  |  |  |  |
| Yes | 466 | 0.585 (0.259-1.325) | 0.199 |  |  |  |
| **Pathologic stage** | 492 |  |  |  |  |  |
| I-II | 402 | Reference |  |  |  |  |
| III-IV | 90 | 1.570 (1.139-2.163) | **0.006** |  | 1.607 (1.163-2.219) | **0.004** |
| **EXOC3L1** | 496 |  |  |  |  |  |
| Low | 245 | Reference |  |  |  |  |
| High | 251 | 1.387 (1.054-1.826) | **0.019** |  | 1.468 (1.113-1.934) | **0.006** |

**Table S4.** Univariate and multivariate Cox regression analysis for OS in KIRP.

| Characteristics | N | Univariate analysis | |  | Multivariate analysis | |
| --- | --- | --- | --- | --- | --- | --- |
|  |  | Hazard ratio (95% CI) | p |  | Hazard ratio (95% CI) | p |
| **Age** | 286 |  |  |  |  |  |
| ≤60 | 133 | Reference |  |  |  |  |
| >60 | 153 | 0.944 (0.519-1.718) | 0.851 |  |  |  |
| **Gender** | 288 |  |  |  |  |  |
| Female | 77 | Reference |  |  |  |  |
| Male | 211 | 0.638 (0.331-1.230) | 0.180 |  |  |  |
| **BMI** | 212 |  |  |  |  |  |
| ≤30 | 136 | Reference |  |  |  |  |
| >30 | 76 | 0.774 (0.344-1.743) | 0.537 |  |  |  |
| **Smoker** | 246 |  |  |  |  |  |
| No | 115 | Reference |  |  |  |  |
| Yes | 131 | 0.557 (0.294-1.055) | 0.073 |  | 0.446 (0.230-0.866) |  |
| **Pathologic stage** | 259 |  |  |  |  |  |
| I-II | 193 | Reference |  |  |  |  |
| III-IV | 66 | 6.548 (3.401-12.608) | **<0.001** |  | 7.421 (3.692-14.913) |  |
| **EXOC3L1** | 288 |  |  |  |  |  |
| Low | 143 | Reference |  |  |  |  |
| High | 145 | 1.542 (0.849-2.801) | 0.155 |  |  |  |

**Table S5.** Univariate and multivariate Cox regression analysis for OS in THCA.

| Characteristics | N | Univariate analysis | |  | Multivariate analysis | |
| --- | --- | --- | --- | --- | --- | --- |
|  |  | Hazard ratio (95% CI) | p |  | Hazard ratio (95% CI) | p |
| **Age** | 510 |  |  |  |  |  |
| ≤45 | 241 | Reference |  |  |  |  |
| >45 | 269 | 774806404.538 (0.000-Inf) | 0.997 |  |  |  |
| **Gender** | 510 |  |  |  |  |  |
| Female | 371 | Reference |  |  |  |  |
| Male | 139 | 1.963 (0.710-5.428) | 0.193 |  |  |  |
| **Race** | 414 |  |  |  |  |  |
| Asian | 51 | Reference |  |  |  |  |
| White | 336 | 74436866.198 (0.000-Inf) | 0.998 |  |  |  |
| Others | 27 | 109860068.891 (0.000-Inf) | 0.998 |  |  |  |
| **Pathologic stage** | 508 |  |  |  |  |  |
| I-II | 338 | Reference |  |  |  |  |
| III-IV | 170 | 7.190 (2.314-22.345) | **<0.001** |  | 4.206 (1.197-14.784) | **0.025** |
| **Histological type** | 510 |  |  |  |  |  |
| Classical | 364 | Reference |  |  |  |  |
| Follicular | 101 | 0.289 (0.038-2.188) | 0.229 |  |  |  |
| Tall Cell | 36 | 0.000 (0.000-Inf) | 0.997 |  |  |  |
| Other | 9 | 0.000 (0.000-Inf) | 0.999 |  |  |  |
| **Residual tumor** | 448 |  |  |  |  |  |
| R0 | 390 | Reference |  |  |  |  |
| R1-R2 | 58 | 3.335 (1.001-11.110) | 0.050 |  | 2.032 (0.574-7.185) | 0.271 |
| **EXOC3L1** | 510 |  |  |  |  |  |
| Low | 255 | Reference |  |  |  |  |
| High | 255 | 0.863 (0.321-2.320) | 0.770 |  |  |  |

**Table S6.** Univariate and multivariate Cox regression analysis for OS in PAAD.

| Characteristics | N | Univariate analysis | |  | Multivariate analysis | |
| --- | --- | --- | --- | --- | --- | --- |
|  |  | Hazard ratio (95% CI) | p |  | Hazard ratio (95% CI) | p |
| **Age** | 178 |  |  |  |  |  |
| ≤65 | 93 | Reference |  |  |  |  |
| >65 | 85 | 1.290 (0.854-1.948) | 0.227 |  |  |  |
| **Gender** | 178 |  |  |  |  |  |
| Female | 80 | Reference |  |  |  |  |
| Male | 98 | 0.809 (0.537-1.219) | 0.311 |  |  |  |
| **Race** | 174 |  |  |  |  |  |
| Others | 17 | Reference |  |  |  |  |
| White | 157 | 1.161 (0.582-2.318) | 0.672 |  |  |  |
| **Pathologic stage** | 175 |  |  |  |  |  |
| I-II | 167 | Reference |  |  |  |  |
| III-IV | 8 | 0.673 (0.212-2.135) | 0.501 |  |  |  |
| **Histologic grade** | 176 |  |  |  |  |  |
| G1 | 31 | Reference |  |  |  |  |
| G2 | 95 | 1.959 (1.007-3.808) | **0.048** |  | 1.861 (0.957-3.618) | 0.067 |
| G3 | 48 | 2.625 (1.304-5.283) | **0.007** |  | 2.741 (1.360-5.522) | **0.005** |
| G4 | 2 | 1.651 (0.211-12.893) | 0.632 |  | 1.476 (0.189-11.538) | 0.711 |
| **Smoker** | 144 |  |  |  |  |  |
| No | 65 | Reference |  |  |  |  |
| Yes | 79 | 1.086 (0.687-1.719) | 0.724 |  |  |  |
| **Alcohol history** | 166 |  |  |  |  |  |
| No | 65 | Reference |  |  |  |  |
| Yes | 101 | 1.147 (0.738-1.783) | 0.542 |  |  |  |
| **EXOC3L1** | 178 |  |  |  |  |  |
| Low | 89 | Reference |  |  |  |  |
| High | 89 | 0.620 (0.408-0.942) | **0.025** |  | 0.592 (0.387-0.906) | **0.016** |

**Table S7.** The 100 EXOC3L1-related genes obtained from GEPIA2.

| Gene symbol |
| --- |
| ENG |
| NOTCH4 |
| CLEC14A |
| FLT4 |
| CCM2L |
| USHBP1 |
| CDH5 |
| ROBO4 |
| ESAM |
| TIE1 |
| TMEM204 |
| ECSCR |
| VWF |
| ARHGEF15 |
| RP11-455O6.2 |
| RAMP2 |
| PCDH12 |
| SLCO2A1 |
| LDB2 |
| PEAR1 |
| SCARF1 |
| BCL6B |
| RP11-389C8.2 |
| GIMAP8 |
| EPAS1 |
| DLL4 |
| HYAL2 |
| EXOC3L2 |
| HID1-AS1 |
| ACVRL1 |
| TMEM255B |
| EGFL7 |
| GPR4 |
| SH2D3C |
| DOCK6 |
| FHOD1 |
| RP11-598F7.5 |
| MTMR9LP |
| HECW2 |
| CXorf36 |
| AC006126.4 |
| HSPA12B |
| TMEM88 |
| RP11-693N9.2 |
| HIGD1B |
| GJA4 |
| CASP12 |
| SLC9A3R2 |
| TEK |
| KDR |
| PLVAP |
| TNS2 |
| GRK5 |
| ARAP3 |
| FGD5 |
| EMCN |
| RAMP3 |
| STARD8 |
| AC005740.6 |
| GIMAP5 |
| AFAP1L1 |
| CD93 |
| SHE |
| RP11-598F7.6 |
| MMRN2 |
| ADCY4 |
| SMAD6 |
| ADGRL2 |
| PECAM1 |
| CASKIN2 |
| ZNF366 |
| RP1-18D14.7 |
| FLT1 |
| HEYL |
| GNG11 |
| RGCC |
| GIMAP7 |
| GIMAP1 |
| NOSTRIN |
| TAOK2 |
| FAM65A |
| LAMB2 |
| ADGRL4 |
| GIMAP6 |
| TBX2 |
| RP4-575N6.5 |
| SOX18 |
| ICAM2 |
| RP1-310O13.7 |
| LINC01197 |
| GALNT18 |
| RP11-236L14.2 |
| RP11-91J19.3 |
| NR2F2-AS1 |
| RP11-674P19.2 |
| CYYR1 |
| KIAA0895L |
| PRX |
| PDGFRB |
| PLXND1 |
